# Advanced medical students’ experiences and views on professionalism at Kuwait University

**DOI:** 10.1186/1472-6920-14-150

**Published:** 2014-07-23

**Authors:** Dalia Al-Abdulrazzaq, Amani Al-Fadhli, Andleeb Arshad

**Affiliations:** 1 Department of Pediatrics, Division of Pediatric Endocrinology, Faculty of Medicine, University of Kuwait, Kuwait City, Kuwait; 2 Department of Pediatrics, Faculty of Medicine, University of Kuwait, Kuwait City, Kuwait; 3 King Saud bin Abdulaziz University for Health Sciences, Medical Education Unit, Al-Hars Al-Watani, Riyadh 14611, Saudi Arabia

**Keywords:** Professionalism, Undergraduate, Curriculum

## Abstract

**Background:**

Professionalism is a core competency in the medical profession worldwide. Numerous studies investigate how this competency is taught and learned. However, there are few reports on the students’ views and experiences with professionalism especially in the Arab world. Our aim was to explore the experiences and views of Kuwait final-year medical students on professionalism.

**Methods:**

This was a questionnaire study of final-year medical students at Kuwait University (*n =* 95). Open- and close-ended questions were used to determine the students’ experiences and views on: definition, teaching, learning, and assessment of professionalism.

**Results:**

Eighty-five of the students completed the questionnaire (89.5%). A total of 252 attributes defining professionalism were listed by our respondents. The majority (98.0%) of these attributes were categorized under the CanMEDS theme describing professionalism as commitment to patients, profession, and society through ethical practice. The most helpful methods in learning about professionalism for the students were contact with positive role models, patients and families, and with their own families, relatives and peers. The students’ rating of the quality and quantity of teaching professionalism in the institution was quite variable. Despite this, 68.2% of the students felt very or somewhat comfortable explaining the meaning of medical professionalism to junior medical students. Almost half of the students felt that their education had always or sometimes helped them deal with professionally-challenging situations. Majority (77.6%) of the students thought that their academic assessments should include assessment of professionalism and should be used as a selection criterion in their future academic careers (62.3%). Most of the students discussed and sought advice regarding professionally-challenging situations from their fellow medical students and colleagues. Seventy-five (88.2%) students did not know which organizational body in the institution deals with matters pertaining to medical professionalism.

**Conclusion:**

This study highlights the influence of the curriculum, the hidden curriculum, and culture on medical students’ perception of professionalism. Medical educators should take in account such influences when teaching and assessing professionalism. Future research should aim at creating a framework of competencies that addresses professionalism in a context suitable for the Arabian culture.

## Background

Professionalism is considered a crucial competency for physicians in all specialties. Therefore, undergraduate and postgraduate programs all over the world, including the Arab world, invest greatly in teaching and assessing medical professionalism in their curricula [1-4].

As professionals, physicians are committed to the health and well-being of individuals and society through ethical practice, profession-led regulation, and high personal standards of behavior [5]. Attributes of a professional physician are not universal and cultural differences exist [6]. Moreover, professionalism is viewed as a social contract between doctors and society [7]. Therefore, it cannot be viewed in isolation from the society as the culture’s influence on physicians and societies may lead to certain differences [6,8]. These differences are being increasingly recognized by medical educators in the Arab world and efforts are being invested in the development of competency frameworks that address professionalism in a manner that is suitable for the local culture such as the development of the Saudi MEDS competence specification for Saudi medical graduates [9].

The Faculty of Medicine at Kuwait University is a rapidly-growing educational establishment founded in 1973 and enrolls an average of 90 students per year. The Faculty’s mission is to promote professional excellence, unfold medical knowledge, blend scholarship and service, follow a path of life-long learning, and share knowledge for the community’s benefit [5]. To accomplish these goals, the Faculty had adopted a system based on case-triggered integrated curriculum for student intake since 2005. This curriculum is primarily influenced by Western curricula from Australia, Europe and North America and is made up of three Phases (I – III). Phase I (1 year) is a pre-professional program consisting of language and sciences courses. Phase II (3 years) consists of system courses that use a variety of methods including a series of problem- based learning (PBL) cases, self-learning activities, didactic lectures, tutorials, laboratory exercises, and hospital visits aimed at stimulating active learning. The Faculty aims at promoting professional and behavioral development through the early introduction of students to the hospital environment at this phase. Phase III (3 years) is a clinical phase consisting of student clerkships through Medicine, Surgery, Community Medicine, Pediatrics, Obstetrics and Gynecology, and Psychiatry. This phase ends up with a pre-internship program that aims at transitioning medical students to being practicing physicians [5]. In this curriculum, the students’ competencies including professionalism are assessed through performance evaluations filled by the medical educators during hospital visits, PBL sessions, tutorials, and laboratory exercises [1].

Western competency frameworks addressing medical professionalism may not be suited to the cultural values of non-Western countries which, similar to Kuwait, had adopted such frameworks [10]. Most of the literature on medical professionalism comes from the West. There is limited data in this area in the Arab world and no data, to date, from Kuwait since the adoption of the new curriculum. Exploring medical students’ views and experiences of professionalism in Kuwait could provide valuable insights on the influence of Western competency frameworks that are being adopted by non-Western cultures. This study also assesses the efficiency of teaching professionalism in the faculty, and identifies any existing deficiencies in the curriculum in teaching professionalism in an Arabian culture. We hypothesize that with the current curriculum the medical students would have a good understanding of the basic and universal fundamentals of medical professionalism, but with potential differences compared to their counterparts in the West. We also hypothesize that the students would have a positive experience with teaching, learning, and assessment of professionalism at the Faculty.

## Methods

This was a questionnaire study carried out among the final-year students in the 2012–2013 academic year. Each student was given a package consisting of two documents. The first document explained the purpose of the study, the voluntary and confidential nature of participation, and included a written consent. The second document was the self-administered questionnaire of the study. The responses of the students were anonymous and not handled by the investigators.

The questionnaire was designed by the authors based on similar studies on medical professionalism among undergraduate and postgraduate students and residents [11-13]. It collected data on demographics (age and gender), the attributes associated with medical professionalism, and the students’ views and experiences with teaching, learning, and assessment of professionalism. The questionnaire asked open- and close-ended questions with responses scaled using the 5-point Likert Scale (Additional file 1).

The questionnaire was initially tested on a pilot of 20 medical students (10 males and 10 females) before it was finalized.

This study was ethically approved by the Joint Committee for The Protection of Human Subjects in Research of the Health Sciences Centre (HSC) and Kuwait Institute of Medical Specialization (KIMS).

### Data analysis

The data were analyzed using SPSS Statistics version 20. The students’ definitions of professionalism were analyzed using Miles and Huberman qualitative data analysis method [14]. A two-step coding system was used to first code the students’ definitions to groups of similar attributes and second to code the emerged groups to three themes defining professionalism according to the CanMEDS roles namely demonstrating commitment to patients, profession, and society through ethical practice; demonstrating commitment to patients, profession, and society through participation in profession-led regulation and demonstrating commitment to physician health and sustainable practice [5]. An extra code was created to account for attributes that were listed by the respondents but did not correctly define professionalism. Quantitative data were analyzed using Chi-Square Statistical Test and a *p* value of < 0.05 was considered significant in the study.

## Results

### Demographics

The overall response rate was 89.5% (85 out of 95 students). There was no significant gender difference among the respondents, where males made up 48.2% (41 students) and females 51.8% (44 students) of the total students’ body in the study. The mean age of the respondents was 23.3 ± 0.831 years.

### Defining medical professionalism

A total of 252 attributes defining professionalism were listed by the respondents. Two hundred and forty seven (98.0%) of these were coded into 18 groups, and categorized under the theme describing professionalism through ethical practice. The three most commonly-listed attributes were punctuality (46 responses), respect (45 responses), and well-attired (30 responses). Only one group was listed under participation in profession-led regulation, namely following regulations (5 responses). No attributes related to commitment to physician health and sustainable practice were listed. There was no significant difference between the responses stated by males compared to females in the study. Only five responses incorrectly defined professionalism and were not included in this analysis (Tables 1 and 2).

**Table 1:** Examples of coding medical professionalism attributes

**Responses**	**Group code**
“*Respect time”*	Punctuality
“*Show respect to seniors, colleagues, and patients”*	Respect
“*Dress appropriately and be well-presented*”	Well-attired
*“Treat people equally”*	Equality
*“Stick to the rules”*	Following regulations

**Table 2:** Students’ definition of professionalism in Kuwait according to the CanMEDS framework

**Professional competencies**	**Attribute group**	**Responses**
		**N (%)**
		**252 (100.0%)**
**Commitment to patients, profession, and society through ethical practice**		247 (98.0%)
	Punctuality	46 (18.2%)
	Respect	45 (17.8%)
	Well-attired	30 (11.9%)
	Ethical	22 (8.7%)
	Knowledge	21 (8.3%)
	Competence	14 (5.5%)
	Care	12 (4.8%)
	Polite	10 (4.0%)
	Hard-working	7 (2.8%)
	Honesty	7 (2.8%)
	Confidentiality	6 (2.4%)
	Equality	5 (2.0%)
	Appreciative	5 (2.0%)
	Responsibility	5 (2.0%)
	Justice	4 (1.6%)
	Skills	3 (1.2%)
	Considerate	3 (1.2%)
	Wise	2 (0.8%)
**Commitment to patients, profession and society through participation in profession-led regulation**	Following regulations	5 (2.0%)
**Commitment to physician health and sustainable practice**	None	0 (0.0%)

### Teaching and learning about medical professionalism

Majority of the students (90.6%) agreed (strongly or somewhat) that medical professionalism can be taught and learned. Students’ responses on ranking methods used in learning about medical professionalism are shown in Table 3. The most helpful methods (rated as most or second most helpful) in learning about professionalism were contact with positive role models, patients and families, and with own family, relatives and peers, whereas books and literature were the least helpful method (rated as the least or second least helpful). There was no significant difference between males and females with regard to these methods.

**Table 3:** Most and least helpful methods to learn about medical professionalism by Kuwait University advanced medical students

	**Method**			
	**Most helpful**			**Least helpful**
	**Contact with positive role models**	**Contact with patients and their families**	**Your own family/relatives and peers**	**Books and literature**
**Student n (%)**	51 (60.0%)	35 (41.2%)	32 (37.61%)	45 (53.0%)

The students’ rating of the quality and quantity of teaching professionalism in the institution was quite variable (Table 4). Despite this, 58 (68.2%) of the students felt very or somewhat comfortable explaining medical professionalism to junior medical students with no significant difference between males and females (Table 4).

**Table 4:** Views and experiences on teaching medical professionalism at Kuwait University

**Question**	**Very adequate**	**Somewhat adequate**	**Undecided/neutral**	**Less than adequate**	**Very deficient**
**Quality n (%)**	3 (3.5%)	30 (35.3%)	6 (7.1%)	28 (32.9%)	18 (21.2%)
**Quantity n (%)**	6 (7.1%)	19 ( 22.4% )	20 (23.5%)	27 (31.8%)	13 (15.3%)
**Question**	**Very comfortable**	**Somewhat comfortable**	**Undecided/neutral**	**Not very comfortable**	**Not at all comfortable**
**Comfortable in explaining professionalism n (%)**	24 (28.2%)	34 (40.0%)	14 (16.5%)	10 (11.8%)	3 (3.5%)
**Question**	**Always**	**Sometimes**	**Every once in a while**	**Rarely**	**Never**
**Degree of encountering a professionally-challenging situation n (%)**	27 (31.8%)	36 (42.4%)	17 (20.0%)	5 (5.9%)	0 (0.0%)
**Usefulness of institution medical education in a professionally-challenging situation n (%)**	10 (11.8%)	34 (40.0%)	22 (25.9%)	16 (18.8%)	3 (3.5%)

Majority of the students encountered professionally-challenging situations in the institution (74.0%). However, only half of the students (51.8%) felt that their education had always or sometimes helped them deal with such professionally-challenging situations (Table 4).

### Assessment and professionalism at Kuwait University

**Figure 1:**
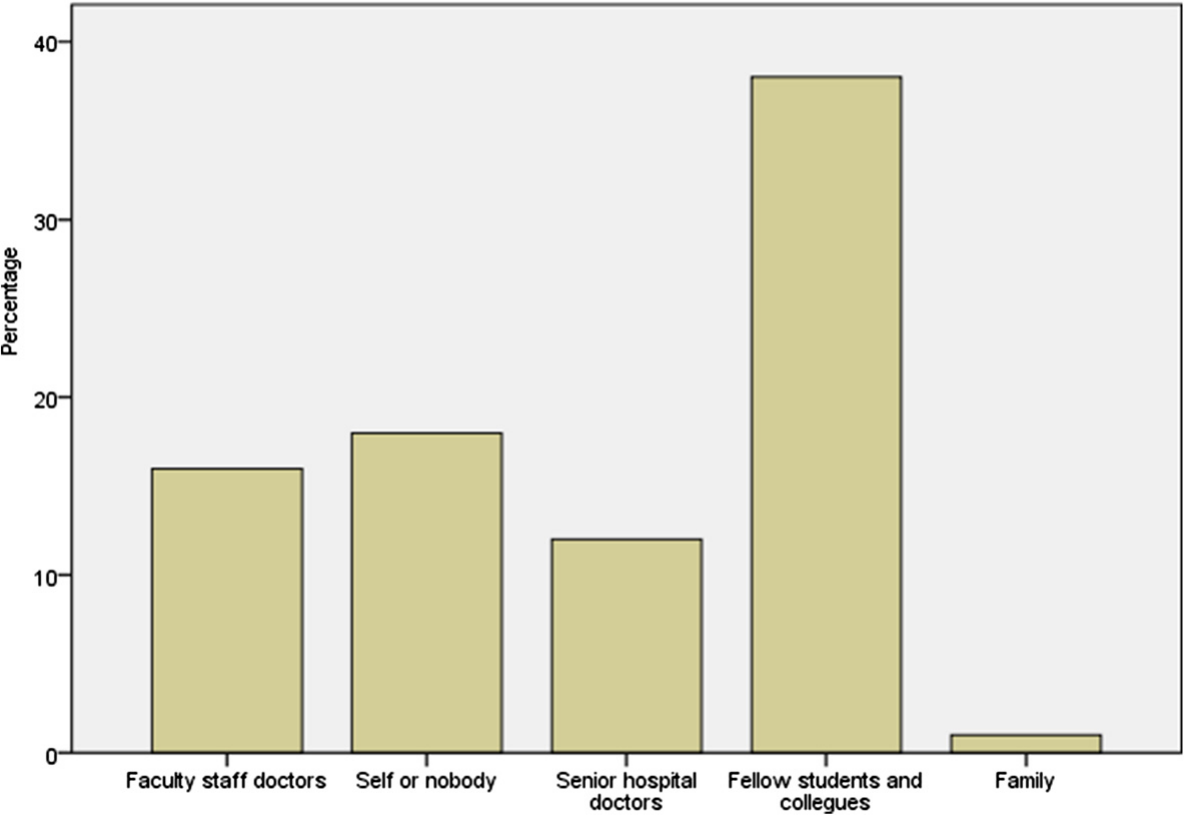
Who do medical students in Kuwait discuss professionally-challenging situations with?

Majority of the students thought that their academic assessments should include assessment of professionalism (77.6%) and should be used as a selection criterion in their future academic careers (62.3%).Most of the students discussed and sought advice regarding professionally-challenging situations from their fellow medical students and colleagues (Figure 1). Lastly, 75 (88.2%) did not know which organizational body in the institution deals with matters pertaining to medical professionalism.

## Discussion

In this study we investigated Kuwait University medical students’ views and experience regarding professionalism. Students in their final year were chosen for the study since they have gone through the full curriculum, thus putting them in the position to assess the impact of the curriculum on medical professionalism.

The CanMEDS roles have been used as a reference to analyze the students’ definitions of professionalism for several reasons. First, the CanMEDS physician competency framework has been adopted by the post-graduate medical education programs in Kuwait [2]. Second, it has been implemented, studied, and adapted around the world [15]. Lastly, this framework describes physician competencies in the medical profession which are expected from advanced medical students in their final year after years of being involved in patient care especially in their pre-internship program in the faculty. There have been efforts in the Arab world to develop a framework of physician competencies suitable for the region, namely the Saudi MEDS [9]. The Saudi MEDS framework was in its early phases of development when this study was conducted and therefore was not used as a reference to analyze the students’ definitions of professionalism.

The students’ definition was heavily focused on describing professionalism through ethical practice. They showed deficiency in viewing professionalism as part of commitment through participation in profession-led regulation and commitment to physician health and sustainable practice (Table 2). Such heavy focus on ethics, in the context of medical professionalism, has also been reported in other studies worldwide [11,16-20]. Firstly, medical students in Kuwait (as in other institutions) spend most of their time in clinical patient care, which involves interaction between the students, the medical team, and the patients. Such interaction constantly involves ethical issues from which students enrich their knowledge about professionalism. There is a clear relationship between the choices and views that trainees make and their daily work [21]. However, the deficiency in viewing professionalism as part of the other two themes clearly reveals lack of student involvement in these two areas of clinical care. Secondly, medical students in this early period of their careers are evaluation-driven. The heavy concentration of ethics in student evaluations at the faculty influences their priorities in defining professionalism. Lastly, this might represent a lack of experience of the medical students in this early period of their careers. This lack of experience, not only of medical students, but also of residents, has been reported in other studies [11,13,17-20].

The three most commonly listed attributes were (in order): punctuality, respect, and well-attired (Table 2). Respect has been identified as an essential attribute identifying ethical practice in other studies [11,17,18,20,22]. Apart from students from Saudi Arabia, who are enrolled in similar medical school systems to Kuwait, punctuality was less recognized in the context of ethical practice in other reports [11,17,18,20]. Moreover, “well-attired” was not part of attributes identifying professionalism in similar studies worldwide [11,17,18,20,22].

Kuwait students’ priorities in attributes linked to ethical practice reveal the role of “hidden curricula” in medical education. The “hidden curriculum” has emerged as an influential concept in professional development [23-28]. It is defined as a set of influences that function at the level of organizational structures and cultures and are outside the formal curricula but play an important role in professional education [29]. Kuwait medical school puts great weight on attendance and personal attire in the students’ evaluations, with subsequent punitive measures, which in turn incorporate these two attributes as priorities in the students’ professional behavior. Unprofessional behaviors in medical schools, which are associated with consequent disciplinary actions influence the students’ subsequent professional growth [30].

Most students in the study believed that professionalism can, indeed, be taught. The two most helpful methods in their learning about professionalism were contact with positive role models, and patients and their families (Table 3). Similar studies showed the same response and state the importance of role models in learning about professionalism [11,17,20]. This supports the notion that professionalism can be learned through contact with others during the daily working life of the trainee [11]. Moreover, such response highlights again the impact of the “hidden curriculum” on learning about professionalism through contact with teachers and patients [25-28].

Different from other studies, Kuwait students found that contact with their own families, relatives and peers is the third most helpful method in learning about professionalism. This highlights the special nature of professionalism as it is significantly related to ethnic and cultural backgrounds regardless of what educational programs the trainees are enrolled in [31,32].This response confirms the existence of cultural variation and context specificity of professionalism that were echoed in other Arabian studies [8,17,33]. The Arabian culture, as many Eastern cultures, tends to be more conservative (emphasizes the maintenance of and respect for social order, tradition, family security and wisdom) [22]. It views the individual in terms of social relationships that maintain harmony by respecting the seniors in one’s family [34]. Furthermore, the socio-cultural background of students in Kuwait is similar to other Arabian cultures, where young adults still live with their close and extended families, even after graduation from high school. From this perspective, it was not surprising that our data showed the influence of families on how students shape their views on professionalism.

It was a cause for concern that the students were variable in rating the quantity and quality of teaching professionalism in the institution (Table 4). Around a quarter to a third felt that the curriculum was not adequate in quality and quantity. Also, while most face professionally-challenging situations in their clinical practice, only half found what is being taught about professionalism helpful in dealing with such situations.

There has been a concern in Kuwait and the Arabian Gulf region about the inadequate emphasis on professionalism in clinical training [35], which may be explained by multiple factors. Medical schools in the region are faced with the challenge of incorporating professionalism in a culturally-appropriate manner. Moreover, the institutions experience a shortage of local teachers and therefore have to recruit them from Western and non-Arab countries with different cultural beliefs and practices. These teachers find it difficult to engage in teaching professionalism in a different culture. Such observation has also been reported from other parts of the Gulf region [17].

Despite the lack of quantity and quality of teaching professionalism, as judged by the students, they were comfortable with teaching this concept to their junior colleagues. The students continue to shape their professional views and behaviors from the “hidden curriculum” and their own family and relatives, and in turn, transfer these views and behaviors to their junior colleagues.

Kuwait medical students, despite their lack of experience in the medical field, recognized that professionalism is a competency that has to be assessed in students’ evaluations and for admission to future career and academic programs. When students need to assess professional behaviors or situations, majority fail to identify institutional bodies (Ministry of Health, Kuwait Medical Association, and the Vice-dean Academic at the Faculty of Medicine) dealing with professionalism in the country. This points, again, to their lack of experience in certain aspects of professionalism that are not part of their daily obligations and roles in the faculty. Such observation is not unique to these medical students and had been demonstrated in the Western literature [11,21]. This also points to the deficiency of the local program in an important aspect of professionalism teaching, which is orienting the students to the appropriate bodies to which they may turn to when confronted with challenges.

### Limitations and strengths

The limitations of the present study include the following. Firstly, we studied the students’ definitions of professionalism using an open-ended question. This may limit their ability to express and define such a sophisticated competency in writing compared to obtaining the definitions through focus groups which provide more elaborate and detailed definitions through discussion. However, such limitation may be a strength at the same time, where each student was comfortable in stating his/her definition in a confidential manner which might be difficult in focus groups. Secondly, we have studied only final-year students, which gives us an idea of what these students had gathered at the end of their education at the faculty, but does not examine the evolution of the students’ perception and experience of professionalism through the different stages of their education. Such comparison between students at different stages of education in the faculty or a longitudinal study will be more informative and is planned by our research team in the near future. Lastly, our results cannot be generalized to all medical schools in the Arab world as variability exists between the curricula in different countries.

Despite such limitations, the study has multiple strengths. It is the first study in Kuwait addressing the issue of professionalism in medical education and one of the few in the Arab world. The results of this study can be generalized to all final-year medical students in the country as we had a high response rate (89.5%) and our medical school is the only one in the country. The results were obtained by anonymous questionnaires that were filled confidentially by the students, thus ensuring honest and non-biased responses. The study examined multiple aspects of medical professionalism giving a more comprehensive analysis of students’ views and experiences in this area. Finally, we used a well-established and validated framework (CanMEDS framework) to examine the students’ definitions of medical professionalism.

## Conclusion

This study gives the first report on medical professionalism as perceived by medical students in Kuwait. This study highlights the influence of the curriculum, the hidden curriculum, and culture on such perception. Medical educators should take in account such influences when teaching and assessing professionalism. We believe that the current curriculum has to be reviewed to concentrate on professionalism as a competency in light of not only ethical practices but participation in profession-led regulations, physician health, and sustainable practice especially for advanced medical students. Moreover, medical educators in the institution should utilize practical methods perceived as helpful to the students in teaching medical professionalism rather than theoretical methods. Future research should move towards exploring the reasons behind cultural differences in the perception of medical professionalism. Furthermore, exploring the views of practicing physicians, other healthcare providers, medical educators, and patients will contribute to a better understanding of medical professionalism especially in a cultural context. Finally, researchers in the region should aim at creating a framework of competencies that addresses medical professionalism in a context suitable for the Arabian culture.

## Competing interests

The authors declare that they have no competing interests.

The authors alone are responsible for the content and writing of this article.

## Authors’ contributions

DA1: Designed the study, contributed to data collection, analysis, and interpretation, and drafted the manuscript. AA2: Contributed to the study design, data collection, and data analysis and interpretation. AA3: Contributed to the study design and revised the manuscript. All authors read and approved the final manuscript.

## Pre-publication history

The pre-publication history for this paper can be accessed here:

http://www.biomedcentral.com/1472-6920/14/150/prepub

## Supplementary Material

Additional file 1 Advanced Medical Students’ Experiences and Views on Professionalism. Click here for file
