# Supplementary material for: Advanced medical students’ experiences and views on professionalism at Kuwait University
Source: BMC Med Educ. 2014 Jul 23;14:150. doi: 10.1186/1472-6920-14-150 (PMC4118198; doi:10.1186/1472-6920-14-150)
Supplement: Additional file 1 — Advanced Medical Students’ Experiences and Views on Professionalism. [file 1472-6920-14-150-S1.doc]

**Additional file 1 Advanced Medical Students’ Experiences and Views on Professionalism**

| 1. List five attributes (phrases/words/explanations) that define Medical Professionalism: 2. Can professionalism be taught and learned? 3. Strongly agree 4. Somewhat agree 5. Undecided/neutral 6. Somewhat disagree 7. Strongly disagree 8. During the time you spent in the Faculty of Medicine at Kuwait University, rank the following methods you have found helpful in learning about professionalism (No.1 being most helpful to No.7 being least helpful)   Contact with POSITIVE role models clinical/faculty teachers  Contact with NEGATIVE role models clinical/faculty teacher  Contact with patients and their families  Your own family/relatives and peers  Lectures and seminars  Videos, movies, or TV shows  Books and literature  Other: specify   1. How do you rate the QUALITY of teaching professionalism in the Faculty? 2. Very adequate 3. Somewhat Adequate 4. Undecided/neutral 5. Less than adequate 6. Very deficient 7. How do you rate the QUANITITY of teaching professionalism in the Faculty? 8. Very adequate 9. Somewhat Adequate 10. Undecided/neutral 11. Less than adequate 12. Very deficient 13. How comfortable are you in explaining the concept and meaning of professionalism to a more junior medical student? 14. Very comfortable 15. Somewhat comfortable 16. Undecided/Neutral 17. Not very comfortable 18. Not at all comfortable 19. To what degree have you encountered professionally-challenging situations (behaviors or conflicts) during your training in the Faculty of Medicine? 20. Always 21. Sometimes 22. Every once in a while 23. Rarely 24. Never 25. How much has your medical education in the faculty helped you deal with professionally-challenging situations (behaviors or conflicts)? 26. Always 27. Sometimes 28. Every once in a while 29. Rarely 30. Never 31. Do you think that assessment of professionalism should be a criterion in your official/formal evaluation? 32. Strongly agree 33. Somewhat agree 34. Undecided/neutral 35. Somewhat disagree 36. Strongly disagree 37. Do you think that professionalism should be a criterion for selection at entry to future career or academic programs? 38. Strongly agree 39. Somewhat agree 40. Undecided/neutral 41. Somewhat disagree 42. Strongly disagree 43. Name the organizational body in the Faculty of Medicine or University of Kuwait that deals with issues regarding professionalism? 44. If you have concerns with a professionally-challenging situation (behavior or conflict) in the hospital or the faculty, whom do you discuss it with? |
| --- |
